# The Innocence Project at Twenty: An Interview with Barry Scheck

**DOI:** 10.1371/journal.pgen.1003692

**Published:** 2013-08-08

**Authors:** Jane Gitschier

**Affiliations:** Departments of Medicine and Pediatrics and Institute for Human Genetics, University of California San Francisco, San Francisco, California, United States of America

The late 1980s witnessed the debut of DNA on the stages of the world's judiciaries. In the United Kingdom, DNA “minisatellites” (discovered by Sir Alec Jeffreys and chronicled previously in this series of *PLOS Genetics* interviews) made their breathtaking appearance in two compelling stories: an immigration case and a double rape-murder case in which a village's entire male population offered up its blood for testing. Meanwhile in the United States, the plodding search for “RFLPs” (restriction fragment length polymorphisms) ultimately gave rise to a set of DNA markers that wended their way into the American courtroom. Directing, producing, and starring in the saga of this transformational technology were a pair of New York lawyers, Barry Scheck and Peter Neufeld, whose relentless efforts and strict adherence to scientific principles have reshaped the landscape of criminal justice far beyond the application of DNA testing.

**Image 1:**
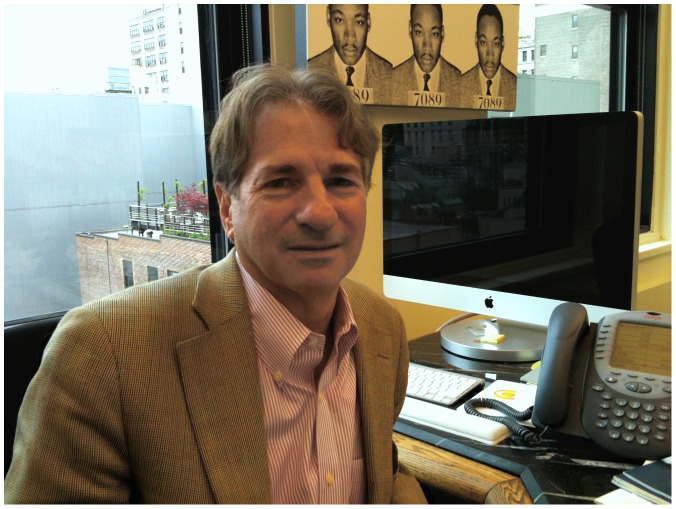
Barry Scheck. Photograph by Jane Gitschier.

Scheck and Neufeld came to know each other in the mid-1970s as public defenders in the Bronx Legal Aid Society. In 1978, Scheck (Image 1) joined Cardozo School of Law to develop law clinics for students in their second and third years, while Neufeld later moved on to private practice. As described in their book *Actual Innocence* (cowritten with Jim Dwyer), Scheck and Neufeld were brought back in to their old Bronx office to assist with the exoneration of Marion Coakley, who had been wrongfully convicted of rape. This case led them not only to learn about DNA as evidentiary material, but also to exonerate other falsely imprisoned individuals using DNA analysis—a mission that became the Innocence Project.

At the time of this interview, the Innocence Project was celebrating its twentieth anniversary, with 290 DNA-based exonerations of innocent people to its credit and, through its network, exonerations of hundreds more through non–DNA-based strategies. Also highly effective in policy making, the Project has championed high standards not just for DNA testing, but also for eyewitness identification, defendant interrogation, and pattern evidence. I was able to meet with Scheck on the seventh floor of 40 Worth Street, home of the Innocence Project. His small corner office, made large by windows overlooking lower Manhattan, was haphazardly decorated with award plaques, clocks, plates, and cups and his desk littered with issues of *Science* strewn among the law journals—all visual reminders of his success in marrying these two disciplines.

**Gitschier:** Let's start with how you came to work at Cardozo Law School.

**Scheck:** I had a colleague in the Bronx Legal Aid Office who got an offer from a new law school. He went to another school, but he told the search committee, “I have a friend who is exactly the kind of person you want.” So they reached out to me and asked me to interview, and this was at the Benjamin N. Cardozo School of Law, which had just started. It is part of Yeshiva University, which also has the Albert Einstein College of Medicine. They were looking for somebody to run all their clinical programs and to be on a tenure track, which clinicians often don't get. And to use the Miller analogy, Cardozo would be to law as Einstein is to medicine. And in fact, I think 34 years later the school is really outstanding, and I've been there from the beginning.

**Gitschier:** Tell me a little about what the clinical programs in law look like.

**Scheck:** The first year of law school is a total immersion process where you train young people to understand legal doctrine; they learn certain analytical skills about how precedent works, how courts work, the difference between legislatures and the judiciary, and they learn common law methods. Basically they understand analytically how lawyers take big problems and put them into very small boxes and categories and manipulate them. So after the first year, the premise is that you're able to get to the more difficult tasks, to be honest with you, which are fact-gathering, problem solving, evaluating institutions, persuasion, ethical issues in terms of dealing with clients and decision makers, and things that arise in that context. That was the intent when I started in 1978.

**Gitschier:** Even though you yourself had only three years in the trenches.

**Scheck:** Well, that was the peculiar part of it. I was lucky enough to get into legal academia at exactly the time that clinical programs were becoming accepted and viewed as a serious enterprise, because those of us that were teaching them on a certain level were not “just practitioners,” but were trying actually to take a serious intellectual dive into these issues. And the big focus was interdisciplinary work. Because the most interesting thing about being a lawyer and being a law professor, frankly, is that you get to learn lots of other disciplines if you so choose, and I so chose. I was very interested in law and psychology, in law and science, and eventually in law and cognitive science, decision-making processes, and of course, persuasion, fact-gathering, and ethics.

After formulating lots of clinics over the years, my friend and colleague Peter Neufeld and I got involved in 1987–1988 with a case from our old public defender's office where we were trying to prove somebody innocent: Marion Coakley. Even though he had an alibi that he was at a prayer meeting with 17 other people, and even though it was apparent that he could be excluded by the blood group substances that were found in semen in a victim of a rape-robbery, there were three eyewitnesses that identified him; therefore he *must* be guilty and he was convicted. Somebody that we had worked with in the serology office of the New York City Medical Examiner's Office, Dr. Robert Shaler, one of the leading lights of forensic science, was on that case. He had just left the serology department to work in a new DNA company called Lifecodes.

So the clinic and I took the case, and I brought Peter in to work with us. Because we knew from the beginning that this would be a very big deal.

**Gitschier:** Why?

**Scheck:** Because we knew that A) getting an innocent person out of jail was a very big deal, and B) Bob Shaler told us that they were going to try DNA testing in this case, and it had never been introduced in an American court.

But what happened was that they tried doing DNA testing on the evidence and they couldn't get results. Ultimately a palm print from the rearview mirror of the car that had been used by the real perpetrator, which had never been analyzed before [as well as other serological evidence from semen], excluded him [Coakley], and he was exonerated.

So then we did a presentation at the Law School, because it was apparent that this [DNA analysis] was a technology that was developed for medical and research purposes, but transferring it to the forensic arena was going to be not so simple.

In fact, one of the things that Peter and I realized was that here were some private companies that were attempting to create market share by promoting their particular form of the RFLP technology, so that everybody would start buying their products and their reagents. The analogy [for the grasp of market share] in those days was betamax vs. VCR—that's how long ago this was. What bothered us, from our law-science backgrounds, was that they really hadn't published their procedures in peer-reviewed journals or done adequate experimentation. And so we were suspicious in terms of the way science and commerce work, that maybe they were rushing into court to capture market share before they had done their homework.

As soon as we made this presentation, where we brought in Dr. Shaler and the late Neville Coleman, who was a serologist and friend, two things happened that were pretty significant. The first was that Mario Cuomo [then governor of New York] asked a criminal justice coordinator named John Poklemba to form a New York State commission to investigate DNA and to see if it could be transferred to the forensic arena. And he put on this commission myself, Peter Neufeld, and a scientist from the Department of Health named Maureen Flaherty, because the New York State Department of Health had long been a model for the country. They also put Dr. Jan Witkowsky on it.

**Gitschier:** Oh, from Cold Spring Harbor.

**Scheck:** Jan is the key to everything! Jan realized that this was going to be very important and decided to hold one of those Banbury Conferences at Cold Spring Harbor. He invited Peter and me to attend, along with a number of other people: Bruce Budowle from the FBI, Rock Harmon who was an Alameda County prosecutor, and most important of all, he invited Dr. Eric Lander.

**Gitschier:** Brilliant man!

**Scheck:** Meanwhile, one of the lawyers who had attended the conference at Cardozo was also from our old public defender's office in the Bronx. He said, “I have the first case where Lifecodes is trying to introduce the DNA evidence in New York. I have a client named Castro, and the prosecution is trying to say that the blood that was found on the defendant's watch was not his blood.” And they also wanted to prove that the blood on the watch came from the victim, the woman who had been murdered. So this lawyer said, “Peter and Barry, you are very interested in this. I don't know what I'm doing. Would you do the admissibility hearing?” Just the hearing on the admissibility of DNA evidence. And we said “Yes” because we wanted to know more about it.

**Gitschier:** So, you were on the side of the defense.

**Scheck:** Yes, but what is important to understand is that we never took the position that the blood on the watch wasn't the defendant's, because there was no reason to say that conclusion was scientifically unsound. It was a clear exclusion: we looked at these RFLP gels and we could see the bands, and they plainly were not going to be the same [as the defendant's].

But when it came to saying that the blood came from the victim and that the odds of this profile in a population were something like one in however many million or more, we were troubled. Because this was exactly at the time when we had our suspicions because Cellmark was competing with Lifecodes and there were all these different entities that were trying to get market share, and they hadn't published yet. And we were wondering whether this technology transfer was really reliable.

So we went to the Cold Spring Harbor Conference with the autorads [of the RFLPs] from the Castro case.

**Gitschier:** Before you did the hearing?

**Scheck:** Before we did the hearing! And we just showed them to Eric Lander. And Eric immediately recognized that there were some extraordinarily serious problems. And, as a matter of fact, one of the people who was at that conference, a wonderful scientist, Dr. Rich Roberts, who was an expert on restriction enzymes and who later won a Nobel Prize, was testifying for the *prosecution*. So we took a look at the data, and everybody naturally assumed, like Dr. Roberts and some other very prominent scientists that had been testifying for these labs, that this ought to work!

**Gitschier:** It *should* work.

**Scheck:** Of course it should work! But, transferring technology from medical and research purposes to the forensic arena is not a trivial exercise.

**Gitschier:** So, the data—these RFLPs from the blood on the watch—did they at first glance match the RFLPs from the victim?

**Scheck:** Well, they were claiming that they did, but when you looked at the gels, what you could see right away was that they had not yet developed what we called then a “matching rule.” In other words, when these fragments were then traveling on the Southern gels, they were literally saying that two bands or alleles that were six standard deviations apart from each other were actually “matches.”

**Gitschier:** Oh, I see, so the data didn't even look great.

**Scheck:** The data looked terrible, and that's why…

**Gitschier:** It wasn't even a population statistical problem…

**Scheck:** Oh no, it was *both*! So what happened was that Eric looked at the data and showed it to Dr. Conrad Gilliam and Dr. Flaherty and they immediately recognized that there were some extraordinarily serious problems.

**Gitschier:** What did Roberts think?

**Scheck:** Well, this is what happened…

**Gitschier:** This is so great—I feel like I'm there at the Banbury Conference!

**Scheck:** Well, you can see all this in a very, very short piece but a very famous one [in *Nature* by Eric Lander]. For forensic DNA, this is like the short article in *Nature* was for the double helix.

**Gitschier:** You are doing another Miller thing!

**Scheck:** All right—another Miller analogy.

**Gitschier:** OK, so the bands looked terrible. They didn't have internally run controls?

**Scheck:** Well they had controls, but they weren't doing the proper experiments, so they weren't making the proper calls. There was a control lane with DNA from one of the scientists—a very good guy actually, so I won't use his name in this—as the human control. But for each of the different probes you'd see five or six bands when you're supposed to see only two, and you'd say, “Well, what was that?” And they'd say, “Oh, it's bacterial contamination.”

And one witness [from Lifecodes] at one point when he was on the stand said, “Well, our scientist was very sick.” And Eric was telling me, “He couldn't be or he'd be septic, he'd be dead!” And then he switched his testimony within probably 45 minutes and said, “No, no, no. The probes were probably contaminated because they were in bacterial vectors.”

So this was a *disaster*! And it was fortuitous. We're talking the transfer of technology from certain applications, medical and research, to the forensic arena, where you're picking up samples covered with “schmutzes,” as we like to call it. It was a technical term in the hearing…

**Gitschier:** Really, that term was used?

**Scheck:** Yes, we used that a lot. As a matter of fact, what happened was that in the middle of the hearing, Eric Lander and the other scientists on our side approached Rich Roberts and the scientists on the prosecution side and said, “This is a mess.” And they acknowledged that yes, this is a mess.

And so this is really historic: the defense and the prosecution scientists got together and wrote a joint statement saying that not only was the matching no good and the molecular biology no good, but also the statistics and the population genetics were not sound. So both sides agreed and they formed a joint statement, after three months of testimony, saying that the match for association was not admissible, and they wrote a whole description of it.

Then they asked the National Academy of Sciences [NAS] to form a commission to look at DNA technology and forensic science in order to make sure that there was a reliable transfer of this technology to the forensic arena. They published a report, “DNA Technology in Forensic Science,” which laid out a lot of bases for how you did experiments and validation studies for all the different technologies. That was very, very influential.

So that's how Peter and I learned molecular biology and population genetics. We learned it from Eric Lander, from Richard Lewontin, from all the various scientists who were involved in this. I don't purport to be any kind of great expert; it's just that we were *there*! What we suddenly realized about all these scientists and these labs is that they were all a few years younger than us but they were all of one generation. And so we were lucky enough to be at the ground floor and understand the implications of this and the potential difficulties.

**Gitschier:** So, what happened to the defendant, Castro?

**Scheck:** Oh, he later pled guilty.

**Gitschier:** So, it was actually a DNA match?

**Scheck:** Oh, I don't know about that, but there was other evidence.

But the Castro case was a big moment. Years later as the Innocence Project began using DNA testing to get innocent people out of prison, something that Peter and I knew from the beginning was that it raised serious questions about all of these *other* forensic science assays that had not been validated with proper science.

**Gitschier:** OK, like what?

**Scheck:** Like what we call “pattern evidence,” such as fingerprints. So we raised this issue, and again the NAS intervened. The NRC [National Research Council] issued a landmark report about three years ago on strengthening forensic science. This report starts with the premise, which the NAS found, that the *only* gold standard forensic science assay that had been properly validated scientifically was DNA testing!

For example, striations on bullets fired from a gun and matching it to a defendant on a crime scene. The expert witnesses look at all the little striations and they go, “I've looked at these under a microscope and, of course, I can't show you the three-dimensional view that I saw, but I can tell you that I think there is ‘sufficient agreement’ and my colleague has looked at it and I can now tell you that this bullet came from this gun to the exclusion of all guns in the universe!”

**Gitschier:** Right.

**Scheck:** So the National Academy committee members asked the ballistics people, “What's your error rate?” “Well, we don't have one.” “What's your measurement error?” “We don't have any.” And they became very upset. And the same thing was true with fingerprints and other pattern evidence.

And so this new National Academy report was in no small measure the product of the Innocence Project urging the National Academy to get involved again, because we saw that DNA exonerations exposed a lot of this inadequately validated science.

This is what has been extraordinary about the Innocence Project: each exoneration is a learning moment for the whole criminal justice system. We've learned a lot about the causes of wrongful conviction. And we were lucky to be there at the very beginning and to be people who were oriented to work in an interdisciplinary way. That's why it's really fitting that it was a clinical program that was the source of the Innocence Project.

**Gitschier:** I first became aware of you through the OJ Simpson trial, so I wanted to ask you, how did you get involved in that? I hadn't appreciated that the Innocence Project had already launched by then.

**Scheck:** It was clear in 1994 that Peter Neufeld and I knew a lot about forensic DNA testing and serology and forensic evidence. And so I was in Madison Square Garden watching a play-off game when we got a phone call from Gerry Uelmen, who was the dean of Santa Clara Law School, and Bob Shapiro saying, “We are representing Mr. Simpson and we're coming up on a preliminary hearing, and there is blood on the crime scene and other forensic evidence. Could you just advise us what questions to ask about technical detail.” So we did that. And then the DNA testing—people don't understand this—in the Simpson case was not completed until February–March, which…

**Gitschier:** …was already into the trial, because the trial started in January 1995.

**Scheck:** January 6^th^.

**Gitschier:** So, you didn't know what the outcome was going to be!

**Scheck:** No, we didn't know what the outcomes or results would be!

**Gitschier:** Who did the testing?

**Scheck:** Well, the FBI, Cellmark, then there was some testing by the Los Angeles crime lab, and the lab was just filled with contamination. And the only silver lining I can see in the OJ Simpson case for the American criminal justice system is that the way that we wound up dealing with the forensic evidence in that case was not to challenge the reliability of DNA testing, but [to challenge] the way that the evidence was handled, because nobody doubts anymore that that was a disaster!

**Gitschier:** So ultimately, you were part of the defense team.

**Scheck:** Well, of course, even giving advice, we were part of the defense team, but slowly and surely, as Johnnie Cochran got involved, we were sort of dragged in to do more and more of the case because it became so long.

But what turns out to be of enormous importance was that one of the prosecutors who did that case, Woody Clark (who is now a judge in San Diego), and I were on a national commission on the future of DNA evidence for the federal government. We turned out a lot of different publications including one on post-conviction DNA testing which was extremely helpful in getting laws in 50 states that there shouldn't be a statute of limitations on DNA evidence, because it is reliable. Also Woody Clark and I and others on this commission were able to issue guidelines: “what every law enforcement officer ought to know about DNA testing,” in effect saying, “learn the lessons from the Simpson case”: never put anything wet into a plastic bag, always change your gloves, and on and on it went. The Simpson case changed the whole way that crime laboratories and evidence gatherers dealt with this evidence. Because you can't have a 21^st^ century technology like DNA testing and 19^th^ century methods of collecting evidence with all the great dangers of cross contamination. It's a “garbage in, garbage out” situation. And people recognized that. That was really a watershed moment.

That was the only silver lining that I can see in the Simpson case in that it changed the entire way that law enforcement approached the gathering of evidence for purposes of DNA testing and frankly for forensic testing generally.

**Gitschier:** So when you say it is the only silver lining…

**Scheck:** It's so many [things]—it was televised, it took…

**Gitschier:** A year of your life…

**Scheck:** It set back race relations in this country. I don't think it did a lot for the esteem that people had for the criminal justice system. There were all kinds of problems that emerged from the saturation coverage.

And of course, the problem that the jury had was that here we had these officers who have told lies, you have scientific evidence that the prosecution brought about itself that maybe was planted. And the timeline had problems. It's very hard to get a jury to agree that he is guilty beyond a reasonable doubt.

The civil trial was different because the burden of proof in a civil trial is by a preponderance of the evidence—more likely than not. And Mr. Simpson had to testify and was cross-examined in the civil trial, and that jury reached a verdict that it was more likely than not that he committed the murders. Now, those two verdicts are not necessarily inconsistent with each other, when you are dealing with quanta of evidence. I think that both verdicts are to be respected. And that's the best I can do.

**Gitschier:** Now I want to close with a little discussion about how the Innocence Project runs.

**Scheck:** So, the Innocence Project…this is a *big* enterprise. There are now a total of 55 projects in the United States and another ten abroad—the Innocence Network. It is also very influential because students graduate. We've been in existence now 20 years. Tomorrow is our gala at the Waldorf Astoria, celebrating 20 years of the Innocence Project.

**Gitschier:** Oh, tomorrow is your anniversary—I'm so lucky to be here today.

**Scheck:** It's an expensive ticket! [Laughter]

We realized about six or seven years ago that what we had to do was become an independent nonprofit entity that was also affiliated with the Law School, in order to grow to an appropriate size. Because right now, as you're sitting in this office, Cardozo Law School is quite a few blocks away. We couldn't find office space near the law school that was large enough to house our entire staff, which is now over 60 people. We have lawyers, paralegals, we have an intake department that assesses all the letters; we still get thousands of letters. We have a policy department that develops policy initiatives in each of the different states where we get eyewitness reform and videotaping of interrogation.

And the one thing we *know* is that there are many more innocent people [in jail] than anybody ever believed. That's what DNA has taught us. I can't give you a precise number, maybe 3.5%, but you are talking about millions of people incarcerated in the US—more than any country in the world!

The Project is a very good institution. It is continually rated as the best-run nonprofit, not because of anything that Peter and I do, but because of our executive director Maddy deLone, and this wonderful staff. We are not paid by the project; I'm a full-time faculty member. And so it's a very efficient, well-run institution that I think is really bringing science-based methods and orientation [to the criminal justice system]. And the greatest ally, as we've reviewed, of the Innocence Project has been the National Academy of Sciences.

The one thing that I've always been surprised about is that, on the one hand, geneticists and the scientific community are very proud of the fact that this wonderful technology has been used so successfully to exonerate the innocent and help identify the guilty, but we haven't raised a lot of money out of the scientific community for this work. So hopefully somebody will read this who has made a fortune in the sciences.

**Gitschier:** Absolutely!

**Scheck:** I think it wouldn't hurt if we got more financial support from the scientific community. So I'd be remiss in this interview if I didn't include that!

